# Fetal membrane imaging and the prediction of preterm birth: a systematic review, current issues, and future directions

**DOI:** 10.1186/s12884-016-1176-5

**Published:** 2016-12-09

**Authors:** Vanessa Nunes, Jennifer Cross, John E. Speich, Danielle R. Morgan, Jerome F. Strauss, Ronald M. Ramus

**Affiliations:** 1 Department of Obstetrics & Gynecology, Virginia Commonwealth University, Richmond, VA USA; 2 Department of Mechanical and Nuclear Engineering, School of Engineering, Virginia Commonwealth University, Richmond, VA USA

**Keywords:** Amnion, Chorion, Ultrasound, Fetal membrane, Preterm birth, Premature rupture of membranes, Preterm premature rupture of membranes, PROM

## Abstract

**Background:**

Preterm premature rupture of membranes (PPROM) is the largest identifiable cause of preterm birth. There is currently no good screening test for PPROM in low-risk asymptomatic patients. Our goal was to identify how imaging methods can be utilized for examining the risks for PPROM in asymptomatic patients.

**Methods:**

This paper is a systematic review of the literature on fetal membrane thickness and its use for the prediction of PPROM. Four key studies are identified and reviewed; two in vitro studies and two in vivo ultrasound studies each using differing methodologies. Additionally reviewed is a study using Optical Coherence Tomography, an emerging technique using near-infrared technology to produce high-resolution images.

**Results:**

There is currently insufficient data to determine the association between fetal membrane thickness and PPROM by ultrasound.

**Conclusions:**

Fetal membrane thickness could have relevant clinical ramifications for the prediction of PPROM. Suggested improvements in study methodology and design will lead to progress in this area of research, as well as the use of newer technologies. Larger sample sizes, histological comparison, uniform methodologies for data collection, longitudinal study design and expanding data analysis beyond fetal membrane thickness to other properties would expand our knowledge in this field. In addition, transvaginal ultrasound should be utilized to improve resolution, as well as emerging methodologies such as MRI fusion imaging using ultrasound and Shear Wave Elastography.

## Background

Preterm premature rupture of membranes (PPROM) complicates approximately 1% of all pregnancies. It is the most significant known cause of preterm birth, accounting for about 30–40% of all preterm births [1, 2]. The perinatal morbidity and mortality associated with preterm delivery is significant, with complications including intraventricular hemorrhage, retinopathy of prematurity, and necrotizing enterocolitis. Complications of PPROM also include sepsis, occurring in 2–20% of affected neonates, with a death rate of 5% [3]. Various etiologic mechanisms have been proposed for PPROM, including membrane stretch, hormonal factors, infection, decreased tensile strength, localized defects, and decreased or altered collagen content in the amniochorion [3]. Cervical length and fetal fibronectin assays have been used to predict the risk of preterm birth, but these methods are generally used to assess women with symptoms or those at high risk of preterm birth [4–8] While some authorities now recommend universal cervical length screening in all patients (including low risk gravidas) this remains controversial, and the efficacy of this approach remains unclear [9]. It is not known how membrane thickness assessment compares to cervical length measurement in a low risk population. Here, we review the literature on ultrasonographic methods used to examine fetal membranes and discuss future opportunities made possible by improved methodologies, transvaginal ultrasound, and emerging imaging technologies.

The basic etiology of PPROM is proposed to involve the interplay between biophysical stresses and biochemical changes leading to rupture of the fetal membranes. Parry and Strauss proposed that the following etiologic factors can result in PPROM [3]:

1. A focal defect in the fetal membranes.
2. Altered membrane structure that results from apoptosis, decreased collagen content, altered collagen structure or increased collagenolytic activity.
3. Altered membrane structure that results from infection and the inflammatory response.
4. Altered membrane structure that results from membrane stretch and polyhydramnios.
5. Altered tensile strength of the fetal membranes.

It has been demonstrated in previous studies that the fetal membranes exhibit viscoelastic properties such as stress relaxation and non-elastic deformation [10–13]. Stress relaxation is a decrease in stress in the membranes when the shape (strain) is maintained, and non-elastic or plastic deformation occurs when tissue remains thinned and does not return to its previous shape when stress is reduced. Tensile strength testing, burst testing, and puncture testing have been used to test membrane strength in vitro [10, 11, 13]. These studies yield a paradigm of how fetal membrane rupture normally takes place and provide key information about fetal membrane structure to guide research on fetal membranes with ultrasonography. Moore and colleagues described the following rupture sequence [13]:

1. Intact fetal membrane distention.
2. Separation of the amnion from the choriodecidua with subsequent rupture of the choriodecidua.
3. Non-elastic further distention of the amnion.
4. Ultimately amnion rupture.

A “zone of altered morphology” is a weak zone of the fetal membranes and is located overlying the lower uterine pole and cervix in term fetal membranes [10, 13–15]. It is assumed that this is the area where rupture occurs [13].

The goal of this review is to understand what is known about fetal membrane imaging and its ability to detect pregnancies at risk for premature delivery. In addition, we wanted to understand how newer imaging technologies could be used to measure membrane thickness. By understanding what is known in this area of research, future studies can be designed that answer the most relevant questions about the relationship between fetal membrane thickness and PPROM.

## Methods

This review started with a comprehensive search to look for manuscripts that evaluated fetal membrane thickness and its potential role in PPROM or preterm delivery. This was done with the help of the medical librarian at our institution. Searches in MEDLINE/PubMed and The Cochrane Library were performed going back to 1990. The medical subject heading (MeSH) terms, keywords, and word variants for “amnion”, “chorion”, “fetal membranes”, “premature birth”, and “ultrasound” were used. The lists of references in relevant articles were also reviewed for additional reports. All searches were confined to English language publications, and excluded animal studies.

Papers identified by these terms were then reviewed by at least two authors to determine if they would help us understand the relationship between fetal membrane imaging and preterm birth. Most manuscripts dealt with the evaluation of fetal membranes in multiple gestations, and were quickly excluded.

After the relevant papers were identified one reviewer (V.N.) extracted important study characteristics using a predesigned data form. Relevant data included: country of origin, timeframe of data collection, number of study subjects and controls, method of ultrasound, gestational age at the time of the study, and a list of study limitations identified by the authors.

While the focus of this investigation was the evaluation of membrane thickness by ultrasound, one additional paper was identified that did not directly address membrane thickness and preterm birth, but did use a novel technology to evaluate the fetal membranes called optical coherence tomography (OCT) [16]. The discovery of this paper led us to proceed with a more general review of newer technologies that have the potential to improve the imaging of fetal membranes. This includes variations of magnetic resonance imaging (MRI) such as MR elastography and fusion imaging, an ultrasound technique called shear wave elastography (SWE), and two optical approaches known as OCT and optical coherence elastography (OCE).

## Results

Overall membrane thickness has been the focus of the published imaging literature on PPROM. A total of four manuscripts were identified that were felt to address this area of interest. These studies were performed between 1996 and 2014. Two studies were conducted in vitro, and two in vivo, yielding inconsistent findings. Table 1 details the key findings, similarities and differences between each paper. Table 2 is a summary of our more general review of novel imaging techniques that may possibly be used to assess the fetal membranes.

**Table 1:** Summary of published studies using imaging technologies to assess membrane thickness

Author	Year	In Vivo or Ex vivo	Sample Size	Description	Results	Limitations
Frigo, et al	1996	Ex vivo	28 (13 primiparas, 15 multiparas) Mean GA 39 1/7 weeks.	Measurements with ultrasound and light microscopy 1 h postpartum. Samples from region of the internal os.	Membrane thickness correlation coefficient by ultrasound vs. histology, *r* = .96, *p* < 0.0001	Limited sample size, term deliveries only, in vitro study design, lack of precise location of membrane thickness measurement for consistency
Frigo, et al	1998	Ex vivo	32 (18 primiparas at 28–32 weeks with PPROM, 11 Cesarean sections, 7 vaginal deliveries, compared to 14 primiparas at 28–32 weeks with intact membranes delivered following induction for chromosomal abnormalities or fetal malformations)	Measurements of fetal membrane thickness with ultrasound and light microscopy 5 cm away from the umbilical cord within 1 h postpartum.	Membrane thickness in PPROM group was thinner, *p* < 0.0001. Correlation coefficient for ultrasonography versus histology was *r* = 0.92, *p* < 0.0001. No significant fluctuations in membrane thickness were observed between 28 and 32 weeks. No differences between vaginal delivery and Caesarean section, nor any significant regional differences. Leukocyte infiltration of the entire amniochorionic membrane was seen in patients with PROM with a significantly thicker intermediary zone, *p* < 0.0001.	Limited sample size, chromosomal abnormality or fetal malformation only in control group, in vitro study design, lack of precise location of membrane thickness measurement for consistency
Severi, et al	2008	In Vivo	158 singletons, transabdominal ultrasound at 18–25 weeks .	Measurement of fetal membrane thickness 3 cm from umbilical cord insertion site.	Women who delivered preterm had a greater membrane thickness than those who delivered at term, *p* < 0.0001. Significant inverse correlation between membrane thickness and gestational age at delivery.	Lack of precise location of membrane thickness measurement for consistency, transabdominal only, fetal membranes only measured once.
Basaran, et al	2014	In Vivo	190 singleton pregnancies	Measurements performed twice, 3 cm from umbilical cord insertion. The first time was between 18 and 22 weeks.	No difference in fetal membrane thickness in the second or third trimester between those who delivered term vs. preterm, *p* = 0.542 for second trimester, *p* = 0.448 for third trimester.	Population with low preterm birth rate (6.8%), lack of precise location of membrane thickness measurement for consistency, transabdominal only. 28 women lost to follow up for second measurement but included in analysis.

**Table 2:** Comparisons of Imaging Technologies

	Definition	Resolution	Previous Studies of fetal membranes
Transabdominal ultrasound	Ultrasound waves	1 mm	Described in text
Transvaginal ultrasound	Ultrasound waves	0.5–1.0 mm	None
MRI (Fusion Imaging)	Real time MRI and Ultrasound synchronized	1.08–1.6 mm	None
Optical Coherence Tomography (OCT)	Near infrared light for real time high resolution cross sectional image of microstructure	1–10 μm	Yes, fetal membranes thicker in full term birth without PROM compared to full term birth with PROM and preterm birth without PROM
Shear Wave Elastography (SWE)	Acoustic radiation that causes displacement, which is used to measure tissue stiffness	10–20 mm	None
Magnetic Resonance Elastography (MRE)	Similar as above using MRI	1.08–1.6 mm	None
Optical Coherence Elastography (photoacoustic technology)	Similar as above using OCT	1–10 μm with displacement measured up to 20 μm	None

Frigo and colleagues published a 1996 paper evaluating fetal membranes in vitro from 28 women who had normal term deliveries [17]. Measurements were carried out on a membrane specimen obtained 5–8 cm from the internal os. The specimens were measured for membrane thickness using a 20-mHz transabdominal ultrasound with a depth of 5–7 mm and an axial resolution of 50–80 μm and lateral resolution of approximately 200 μm. These measurements of thickness were then compared to those of the same specimen using a light microscope on 250X magnification. The findings of the in vitro ultrasonographic examination were highly correlated to the histological measurements, with a thickness of 0.83 mm ± 11 mm (0.72–1.08 mm) on ultrasound, and mean measurements of 0.82 ± 0.13 (0.71–1.10 mm) by light microscopy, with a correlation coefficient of *r* = 0.96, *p* < 0.0001. No intra-observer variability or inter-observer variability data was included in this study. These authors note a variation coefficient of 0.5%–1%. The authors were able to visualize the amnion and chorion as echo-dense, the intermediary zone as echo-poor and inhomogeneous with the boundaries of each zone easily discernible. The authors indicated that the intermediary zone appeared to be primarily responsible for membrane thickness [17]. This intermediary zone lies between the amnion and chorion, and it allows membranes to absorb physical stress by providing a surface for which the amnion can slide on the chorion. It is primarily made of type III collagen, hydrated proteoglycans and glycoproteins.

In 1998, in a second study, Frigo and colleagues extended their first study by comparing, in vitro, the thickness of the amniochorionic membrane from 18 patients with PPROM and 14 control samples [18]. Of note, all the patients in the control group had intact membranes and were undergoing induction due to a chromosomal abnormality or fetal malformation. The inclusion criteria were primiparity, age 18–25, gestational age 28–32 weeks, and maximum weight gains during pregnancy of less than 15 kg. Exclusion criteria were preeclampsia, obesity, smoking, alcohol or other drugs, and maternal metabolic disorders. A different protocol was employed as in their previous study using a specimen located 5 cm from the umbilical cord. A 20-MHz transabdominal ultrasound probe was used, with 5–7 mm penetration, axial resolution 50–80 um and lateral resolution 200 μm. Measurements using this probe were compared to light microscope measurements at 250X magnification. They also examined histological outcomes such as structure, cell typology, and morphology as well as leukocyte invasion in each of the layers. Linear regression analysis and correlation coefficients were used for statistical analysis. The PPROM group had a membrane thickness of 0.54 ± 0.9 mm which was statistically significantly different compared to the control group with a membrane thickness of 0.74 mm ± 1.01 mm, (*p* < 0.0001). No intra-observer variability or inter-observer variability data was included in this study. Similar to their other study, there was an increased thickness in the intermediary zone of the placental membrane [17, 18]. Distinct leukocyte infiltration of the entire amniochorionic membrane in patients with PPROM was noted on histologic preparation. The intermediary zone was thicker in PPROM, measuring 0.14 mm ± 0.07 versus 0.11 mm ± 0.04 (*p* < 0.0001). There were no statistically significant differences in membrane thickness within the groups between gestational ages 28 to 32 weeks. There was also no statistical difference between the sonographic and histologic measurements. The measurements were found to be identical, with a correlation coefficient of r =0.92, (*p* < 0.0001). These authors noted that although previous studies found a discrete rupture zone, they “did not detect significant regional differences in membrane thickness, which was in fact the same at all measuring points.” This group hypothesized that two different mechanisms are responsible for PPROM: ascending infection that causes the differences in the first and early second trimester, and increased mechanical strain on the lower uterine segment in the late second and third trimester.

In a 2008 study, Severi and colleagues examined 158 consecutive singleton pregnant women with ultrasound evaluations between 18 and 35 weeks of gestation [19]. These women were cared for at a referral center for high risk pregnancies, but the investigators do not provide information on the nature of their pregnancy complications (except to say that cerclage, previa, and fetal anomalies were exclusions). A 2.5–6.6 MHz transabdominal probe was used and the region of interest was captured in vivo at about 3 cm from the umbilical cord insertion. This image was magnified to occupy >75% of screen before measurements were taken. The measurements of fetal membrane thickness of those that delivered preterm were then compared to those that delivered at term. There were no statistically significant differences between the two groups at baseline. The mean thickness was 1.67 mm ± 0.27 mm for those that delivered preterm versus 1.14 mm ± 0.30 mm (*p* < .0001) for those that delivered at term. The inter-observer variability was 6.7% and the intra-observer variability was 6.5%. In this study, there was a statistically significant and inverse correlation between membrane thickness and gestational age at delivery (*r* = −0.302, *p* −0.0001) as well as a statistically significant inverse correlation between fetal membrane thickness and time elapsed between measurement and delivery (*r* = −0.306, *p* < 0.0001) [19]. A cut off for the best value of membrane thickness to predict preterm birth was chosen by a receiver-operating characteristics (ROC) curve analysis. The cut-off value of 1.2 mm had a sensitivity of 100% (95% CI, 80.3–100%) and specificity of 69.5% (95% CI: 61.2–77.0). The preterm birth rate in this population was 10.8%. This study found that thickened membranes could precede preterm delivery by up to 70 days. This duration presents an important opportunity to consider intervention if this data paradigm could be further validated. When the sonographic thickness was below the cut-off premature delivery did not occur, even in women experiencing contractions. The authors of this study also suggested that measuring membrane thickness near the cervix may be more indicative of an ascending infectious process, and may be better suited to evaluation by transvaginal ultrasound [19].

The most recent relevant study examining fetal membrane thickness by ultrasonography is a 2014 study by Basaran and colleagues. It examined amniochorionic membrane thickness in prediction of preterm birth among an asymptomatic pregnant patient population [20]. This study examined 190 women and measured the fetal membranes between 18 and 22 weeks and then again between 28 and 32 weeks using transabdominal ultrasound. A 5.7 MHz abdominal convex probe was used, employing a method similar to that in the Severi study [19, 20]. This team did not find a statistically significant difference between fetal membrane thicknesses in the second or third trimester compared to those that delivered pre-term. Term patients had statistically significant thicker membranes between the first and second measurements (*p* < 0.0001) whereas there was no statistically significant increase in the membrane thickness of the pre-term group. Second trimester measurements were 0.790 mm ± 0.230 mm in the term deliveries and 0.770 mm ± 0.270 mm (*p* = 0.542) with preterm birth. Third trimester measurements were 0.880 mm ± 0.270 mm in the term group and 0.910 mm ± 0.200 mm in the preterm group (*p* = 0.448). The inter-observer variability coefficient was 0.48 and the intra-observer variability coefficient was 0.55. The preterm birth rate in this population was 6.8%. These authors noted that the lower rate of preterm birth in this study might have contributed to the differing results from the Severi and colleagues study [19, 20]. These authors also suggest that magnification artifacts that occur during the still image as well as differing etiologies of preterm birth may contribute to the differences in their results compared to that of Severi.

These studies measuring fetal membrane thickness with ultrasonography are methodologically limited and have yielded inconsistent results. The first study by Frigo found that transabdominal ultrasound yielded in vitro measurements of membrane thickness that were similar to that of histological measurements [17]. The second study by Frigo found that in vitro fetal membrane thickness was significantly thinner in PROM patients with evidence of leukocyte infiltration and a thicker intermediary zone [18]. The two in vivo studies used similar ultrasound methodology but found conflicting results [19, 20]. Severi found statistically significant thicker membranes in those that delivered preterm whereas the Basaran study found no statistical difference [19, 20]. The characteristics of fetal membranes after delivery may reflect changes to the fetal membranes that occur during or after delivery. With respect to PPROM, given the high rates of infection and chorioamnionitis, the fetal membrane changes may be incorporating those changes specific to the mechanisms of infection and inflammation [21, 22]. These studies differ from each other in that they included different preterm birth rates, different sample sizes, ex vivo versus in vivo study design, transabdominal ultrasound, differences in gestational age at fetal membrane measurements and differing inclusion and exclusion criteria. For example the Frigo 1998 study uses fetal membranes from those that delivered at term due to a major fetal anomaly whereas both in vivo studies by Severi and Basaran specifically exclude those with major fetal anomalies [18–20]. The study by Severi found a preterm birth rate of 10.8% and found a statistically significant difference in birth outcomes for those with membranes thicker than 1.2 mm [19]. In contrast, Basaran used a similar methodology with a much lower preterm birth rate of 6.8% and did not find a statistically significant difference between fetal membrane thickness in those who delivered preterm and those who delivered at term. [20] Frigo and colleagues found preterm birth associated with thinner fetal membranes with ex vivo ultrasound measurements, while the in vivo studies by Severi and colleagues had the opposite finding, albeit measuring thickness at different locations [17–20]. Basaran and colleagues note that a reason for the differing results of these two in vivo studies are that PPROM represents a final common pathway for a variety of etiological mechanisms. [20] This may mean that a factor such as infection or inflammation may lead to thicker membranes whereas another factor may lead to thinner membranes. A possible solution offered by Basaran is to investigate the changes of the membranes over time, those that increase in a statistically significant manner as their study suggested would fit a “term pattern” [20].

## Discussion

A question one must ask is about the clinical relevance of this systematic review. We have rigorously examined the small body of literature of on fetal membrane measurement for the prediction of PPROM, and the studies reviewed here have demonstrated that fetal membranes can be measured in vivo using transabdominal ultrasound, that it can be conducted in the second or third trimester and that individual components of the fetal membranes can be identified.

However, the in vivo studies have provided conflicting evidence as to whether or not thicker fetal membranes are associated with term or preterm birth.

Membrane thickness assessed by transabdominal ultrasonography has been the focus of the existing ultrasound literature on PPROM. The limitations of transabdominal ultrasound in this setting are numerous. The probe has a maximum penetration at which the resolution begins to decline. Furthermore, obesity is rising to epidemic proportions with growing numbers of pregnant women having a BMI > 30. It is more difficult to perform transabdominal ultrasound on these women, which limits the utility of this approach for predicting the risk of PPROM. Transvaginal ultrasonography can provide a higher resolution and higher magnification to show differences in structure not previously seen by abdominal sonography. With the transvaginal ultrasound probe having a closer proximity to the cervix, it can provide a higher resolution image compared to a transabdominal ultrasound image. Case reports have described the use of Doppler to detect amniotic fluid leakage and the “moon sign” of the chorion separating from the decidua at the internal os can be detected as well with transvaginal ultrasound [23, 24].

There is promise in the areas of transvaginal ultrasonography, shear wave elastography and optical coherence tomography for the measurement of fetal membranes in vivo with current clinical applications. These technologies will be discussed in detail next, and other technological approaches not currently available in clinical practice are included in this review for completeness. In short, MRI, MRI-ultrasound fusion imaging and transvaginal ultrasound offer a higher resolution to take static measurements, and shear wave elastography allows for the measurement in vivo of viscoelastic properties that provide a more functional assessment of the fetal membranes.

### MRI

MRI studies have been limited and have not been used directly to measure the fetal membranes. After an extensive literature review, the most relevant study found used the advantages of MRI over ultrasonography to study amniotic sheets. The characteristics cited by Kato and colleagues in 2004 are a wider field of vision, improved tissue contrast and examination of the posterior uterus (that is normally hindered in the third trimester by the fetus) [25]. According to this study, compared to ultrasonography “MRI is more fitted to understand whole uterine structures by the 3-D observation”, perhaps this advantage can be used to measure the fetal membranes [25]. A newer technologic derivation of MRI, named “fusion imaging” could provide the advantages of MRI with the real time and added Doppler technology that are present with ultrasound. With this advancement, real time ultrasound images are synchronized with MRI images and have been used in the past for prostate cancer biopsy and hepatocellular carcinoma detection. In this study, Fusion Imaging is used for prenatal diagnostic purposes especially in situations of “unfavorable fetal position, advanced gestational age, multiple pregnancy and maternal obesity” and a resolution for invasive procedures up to 1.08 mm to 1.6 mm which would be helpful on the scale of something as thin as the fetal membranes [26].

### Shear wave ultrasonography

Shear Wave Elastography (SWE) is used to examine the viscoelastic properties of tissues. It has been used in the examination of breast, thyroid and liver masses and for measuring cervix elasticity, placental insufficiency and in studying the myometrium during labor [27–29]. SWE uses acoustic radiation to generate a shear wave which is a cone-shaped force that is measured using high velocity ultrasound at around 5000 to 20,000 KHz [28]. This measurement of the shear wave, also known as Shear Wave Speed (SWS), is then estimated by software in meters per second and is faster in hard or stiff tissue and slower in softer tissues. SWE has been used to evaluate the cervix, finding differences in the shear wave speed between cervixes of differing lengths and after treatment with prostaglandins for cervical ripening [28]. The placenta has been examined as well to examine the elastic modulus of the placenta in comparison to other established measures of placental perfusion and is found to have potential for measurement of placental function [27]. Of note, a similar study on the myometrium using SWE to measure uterine contractions during labor used an elastogram window of 18 by 15 mm squared [29]. In another study, the resolution of ultrasound elastography has been reported to be as low as hundreds of microns, though it is not clear if this is possible in vivo [30]. As discussed previously, the fetal membranes are approximately 1 mm thick, and it is unclear whether or not the resolution of SWE technology could be adapted to this thickness.

### Optical coherence tomography

Optical Coherence Tomography (OCT) is an emerging technology that can be used for measurement of the fetal membranes. This technology is unlikely to be used in vivo clinically at this time. OCT uses near-infrared light to produce real-time high resolution (1–10 microns), cross-sectional images of in vivo tissue microstructure [16]. This technology achieves those advantages while avoiding some of the challenges of x-ray crystallography in that it does not require cytotoxic fixation, tissue removal or ionizing radiation and has the potential advantage of imaging internal organs when used with endoscopes or catheters [16]. A study from 2012 examined thirty-six fetal membranes and compared the thickness by histological measurement and light microscopy in nine fetal membranes. The authors then compared the membrane thickness by birth outcome to either normal birth, preterm birth without premature rupture and full term birth with premature rupture of membranes [16]. The results revealed that OCT did not detect statistically significant differences between birth types at the same locations; however the normal birth membranes were thicker than the preterm birth membranes in all locations [16]. Histological analysis was found to be more accurate than OCT due to the lower resolution of OCT. Future work can continue to increase the resolution of OCT and to standardize the preparation for OCT versus standard histological preparation.

### Magnetic resonance elastography

Magnetic Resonance Elastography (MRE) was first described in 1995 [31]. It uses technology similar to ultrasound-based elastography and has been employed in studies of the liver, heart, lung, breast, cervix and uterus. This technology is unlikely to be used in vivo clinically for fetal membrane measurement in the near future. According to a study by Liang and colleagues in 2014, the resolution of MRE is at a millimeter, which is the approximate thickness of the fetal membranes [30]. The literature on MRE of the breast is interesting in that it describes the development of a compressive MRE and a non-compressive breast MRE. In the compressive model, an external force is applied to the breast one at a time and causes a shear wave in the breast, which is read by a motion sensitive MRI sequence and stiffness is then computed [31]. In the other model, the MRE driver applies the force through the chest wall rather than on the tissue itself avoiding the complicating effect of compression on stiffness measurements. Both of these techniques could be applied to the fetal membranes. In comparison with ultrasound elastography of the breast, MRE technology has proven more advantageous. Strain imaging of the breast or qualitative ultrasound elastography has been improved on by MRE by having more contrast to surrounding tissue due to a better image [31]. Stiffness imaging or quantitative elastography has been improved by MRI due to improved imaging of deep tissue. MRI of the uterus has been used to measure the stiffness of fibroids, but this is not as relevant to the elastography of the fetal membranes. MRE of the cervix has been described by Jiang and colleagues in 2014 in which they used 3D multifrequency Magnetic Resonance Elastography (3DMMRE) to measure differences in viscoelastic properties of the uterus and cervix throughout the menstrual cycle [32]. This has improved upon sonographic elastography by providing a viscoelastic map of the uterus and cervix. This technology could thus be applied to make a similar map of the fetal membranes and possibly predict the “zone of altered morphology”.

### Optical coherence elastography

Optical Coherence Elastography (OCE) is similar to ultrasound elastography and MRI elastography but it has expanded the resolution of those modalities. Compared to ultrasound elastography and MRI elastography, OCE is still an emerging field and is not yet ready for clinical use. OCE is reported to have a resolution of several microns, which is resolution at the cellular level. A 2014 study by Ford and colleagues examined the in vitro cornea utilizing OCE to measure displacements at the level of 20 microns, the level of the collagen micro-structure [33]. This is important to the fetal membranes because collagen cross-linking and altered morphology therein has been described as a possible precursor to PPROM. The depth of penetration of OCE is limited to 1–2 mm, this limits its use in vivo. It is postulated that using a catheter or other probe would prove useful for extending this research to the cervix.

#### Future directions

**Fig. 1:**
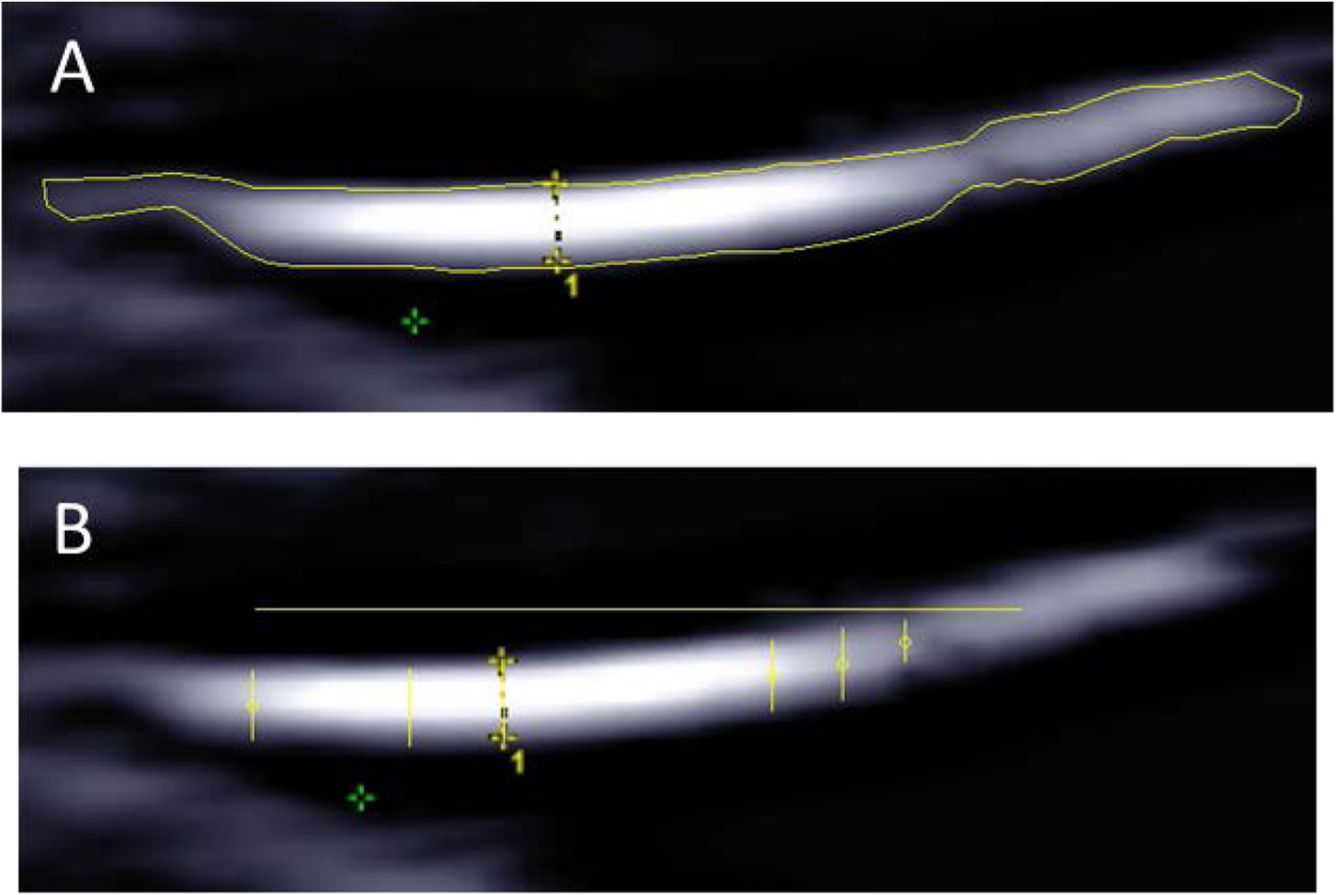
Ultrasound image of fetal membranes at 21 weeks 4 days, obtained transvaginally. **a** An outline was manually drawn around the membranes to determine the area and length, which were used to approximate the average thickness using the equation: average thickness = area/length. **b** Six thickness measurements were performed along a 1 cm range near the center of the image of the membranes (1 cm scale bar along the top) to identify the maximum and minimum thicknesses. Values are provided in Table 3

The existing body of literature on fetal membrane thickness measurements is limited to two in vivo studies and two in vitro studies [17–20]. The findings are inconsistent and other studies are needed to better examine whether or not this is a viable method of predicting PPROM and its associated perinatal outcomes. In this section, we explore suggestions of future directions for study design that could improve upon the existing, very limited body of literature. First, we recommend expanding sample sizes to include a larger number of asymptomatic controls as well as a higher risk of preterm birth population. Second, we recommend comparing transabdominal and transvaginal ultrasound measurements of each subject over time. This would provide a longitudinal analysis with the added resolution of transvaginal ultrasound. The imaging assessments that should be used to predict PPROM include both dynamic and static measurements. Dynamic assessment would involve imaging fetal membranes at multiple time points in pregnancy, and quantifying these changes over time. Measurements should then be repeated throughout pregnancy and should include: membrane thickness, viscoelastic properties at rest and in response to pressure. Static assessment should be a single study performed at a defined gestational age with evaluation of the membrane characteristics noted above such as evidence for focal separation of the amnion and choriodecidua, or rupture of the choriodecidua. Measuring fetal membrane thickness should be performed via transvaginal and transabdominal ultrasound from 18 to 24 weeks gestation. Third, we recommend including histological comparison to the ultrasound measurements. Jabareen and colleagues have developed a histologic protocol for comparison of fetal membrane thickness [11]. El-Khwad and colleagues have developed a protocol in which the cervical region of the fetal membranes can be stained with Gentian Violet introduced via the vagina that will provide a marker for the cervical region of the fetal membranes to be histologically examined [10]. Fourth, we recommend measuring subjects that are undergoing elective Cesarean section before delivery using transabdominal and transvaginal fetal membrane measurements and comparing those to a histologic protocol. Fifth, we recommend expanding the measurements of the fetal membranes beyond the single thickness measurement. Other measurements including maximum thickness, minimum thickness, area, and average thickness are easy to obtain via image analysis software. As an example, Fig 1 shows an ultrasound image of fetal membranes at 21 weeks 4 days. The image was post-processed using Digimizer software (MedCalc Software) to provide the measurements in Table 3. In Fig 1a, an outline was manually drawn around the membranes and the software provided the area and length of this region. The average thickness was approximated as area/length. In Fig 1b, six thickness measurements were performed along a 1 cm range near the center of the image of the membranes to identify the maximum and minimum thicknesses in that region. Sixth, we recommend utilizing emerging technologies such as Shear Wave Ultrasonography or MRI in feasibility studies.

**Table 3:** Fetal membrane geometry from the image in Fig 1

Thickness measured in the clinic	0.09 mm
Area	14.5 mm^2^
Length	16.46 mm
Average Thickness	0.88 mm
Minimum Thickness	0.55 mm
Maximum Thickness	1.09 mm

The available options for imaging the fetal membranes have increased considerably and there are now high-resolution options such as MRI and Optical Coherence Tomography that can better visualize fetal membranes. There are also now options such as Shear Wave Elastography, Magnetic Resonance Elastography and Optical Coherence Elastography that can be used to possibly measure the elastic properties of the membranes. Transvaginal ultrasonography can also be used to measure the regions of the fetal membranes closer to the cervix. If these studies are combined with improved methodologies such as larger sample sizes, this area of inquiry could prove fruitful. An ideal study of this area would combine the various imaging technologies, histologic comparisons and a larger study and control population that can be followed over time with birth outcomes compared to imaging findings.

## Conclusions

Utilizing improved study methodology through larger sample sizes, longitudinal analysis and improved data analysis as well as transvaginal ultrasound, the area of fetal membrane imaging to predict the risk of preterm birth has exciting potential. If successful this screening process would be easy to add to the imaging studies that are already standard of care for both high-risk and low-risk pregnancies. Furthermore, emerging technologies such as shear wave elastography, optical coherence tomography and fusion MRI imaging hold the promise of improved examination of the fetal membranes. Along with advances in ultrasound technology, future studies may be able to identify characteristics of the fetal membranes that are predictive of PPROM; such a finding would help us better understand this important cause of preterm birth and possibly help clinicians mitigate its associated morbidity and mortality.
